# Role of astrocytes in manganese mediated neurotoxicity

**DOI:** 10.1186/2050-6511-14-23

**Published:** 2013-04-18

**Authors:** Marta Sidoryk-Wegrzynowicz, Michael Aschner

**Affiliations:** 1 Department of Pediatrics, Vanderbilt University Medical Center, Nashville, TN 23233, USA; 2 Department of Pharmacology, the Kennedy Center for Research on Human Development, and the Center for Molecular Toxicology, Vanderbilt University Medical Center, Nashville, TN 23233, USA

**Keywords:** Astrocytes, Manganese (Mn), Neurodegeneration, Transporter, Glutamine (Gln), Glutamate (Glu), Neurotransmission, PKC signaling, Ubiquitin-mediated proteolytic system

## Abstract

Astrocytes are responsible for numerous aspects of metabolic support, nutrition, control of the ion and neurotransmitter environment in central nervous system (CNS). Failure by astrocytes to support essential neuronal metabolic requirements plays a fundamental role in the pathogenesis of brain injury and the ensuing neuronal death. Astrocyte-neuron interactions play a central role in brain homeostasis, in particular *via* neurotransmitter recycling functions. Disruption of the glutamine (Gln)/glutamate (Glu) -γ-aminobutyric acid (GABA) cycle (GGC) between astrocytes and neurons contributes to changes in Glu-ergic and/or GABA-ergic transmission, and is associated with several neuropathological conditions, including manganese (Mn) toxicity. In this review, we discuss recent advances in support of the important roles for astrocytes in normal as well as neuropathological conditions primarily those caused by exposure to Mn.

## Manganese: general characteristics and toxicity

Mn is an essential trace metal, which is commonly found in the environment. Mn is present in all tissues and is required for the maintenance of proper function and regulation of numerous biochemical/cellular reactions [1]. Mn is an integral component of multiple enzymes, including glutamine synthetase (GS), mitochondrial superoxide dismutase (SOD), arginase and pyruvate carboxylae [2]. On the other hand, in humans excess deposition of Mn in the central nervous system (CNS) leads to neurological abnormalities, referred to as manganism [3]. Manganism is characterized by variety of psychiatric, cognitive and motor disturbances that resemble those inherent to Parkinson’s disease (PD) [4]. However, the primary brain regions targeted by Mn are the globus pallidus and striatum of the basal ganglia, whereas neurodegeneration in PD is predominantly confined to the substantia nigra [5].

In the early stages of manganism, patients display psychotic symptoms, which progress to chronic disturbances in extrapyramidal circuits, leading to postural instability, dystonia, bradyskinesia, micrographia, mask-like facial expression and speech disturbance [6-9]. Mn toxicity is a potential occupational health hazard in workers in ferroalloy plants, automotive repair technicians, battery manufacturers and welders [10-12]. Increased Mn levels in serum have been noted in chronic liver failure as a result of the inability to excrete the metal *via* the biliary system [13]. In addition, Mn pollution has been a subject of environmental concern because of consumption of contaminated water containing high levels of the metal as well as exposures from soy-based infant formulas and total parenteral nutrition [14]. Health risks associated with Mn exposure have also been associated with organic Mn-containing pesticides, such as Mn ethylenebis-dithiocarbamate [15]. An organic Mn compound methylcyclopentadienyl manganese tricarbonyl, a Mn derivative, is used as an antiknock agent in automobile fuels [16].

Mn transport within the CNS is mediated by several transporter proteins as a free ion or a non-specific protein-bound species [17]. In the 3^+^ oxidation state Mn binds to the transferrin receptor (TfR) and its transport competes with iron (Fe) [18]. Transport of Mn in the 2^+^ oxidation state is mediated by the family of natural resistance-associated macrophage proteins (Nramp), the divalent metal transporter-1 (DMT-1) [19,20]. Moreover additional channels/transporters have been identified as Mn-transporting carriers. These include the divalent metal/bicarbonate ion symporters ZIP8 and ZIP14, various calcium channels, the solute carrier-39 (SLC39) family of zinc transporters, park9/ATP13A2, the magnesium (Mg) transporter hip14 and the transient receptor potential melastatin 7 (TRPM7) [21], to name a few.

The mechanisms of Mn neurotoxicity are not completely understood. Oxidative/nitrosative stress (ONS) has been implicated in Mn-induced adverse effects [22,23]. Mn preferentially enters the mitochondrial matrix *via* the calcium (Ca^2+^) uniporter [24]. It has slow clearance from mitochondria leading to increased matrix Ca^2+^ and reactive oxygen species (ROS) generation [25]. Mn also affects the antioxidant system by depleting glutathione (GSH) and glutathione peroxidase [26]. In addition, activation of oxidative stress-sensitive kinases and transcription factors, including nuclear factor- NF-κB has been implicated to mediate the neurotoxicity of Mn [27,28]

### Astrocytes in the central nervous system function and dysfunction

#### General role of astrocytes in brain function: interaction with neurons

Astrocytes are key regulators in brain function, characterized predominantly via their close interaction with neurons. Astrocyte metabolism, including energy generating pathways and amino acid homoeostasis is tightly coupled to that of neurons. This part of the review will discuss aspects of astrocyte metabolism which are involved in regulating key neuronal functions.

The metabolic relationship between astrocytes and neurons is critical for energy metabolism as well as for the synthesis of neurotransmitters [29]. Neurons are dependent upon astrocytes since they lack the enzyme pyruvate carboxylase (PC) and therefore dependent on astrocytes for *de novo* synthesis of glutamate (Glu) as well as for replenishment of Krebs cycle intermediates [30-32]. Moreover, Glu is not efficiently transported across the blood–brain barrier; thus astrocyte derived glucose serves as a precursor for synthesis of this neurotransmitter [33], and maintenance of optimal Glu levels require astrocytic support [34].

A major portion of astrocytic Gln is critical for maintaining Gln supply to Glu-ergic terminals as well as for generating neurotransmitters. Gln released from astrocytes *via* the bi-directional functioning amino acid systems N and ASC is taken up into neurons by the unidirectional system A [35,36]. In neurons Gln is metabolized to Glu, generating indirectly γ-aminobutyric acid (GABA) as well as the tricarboxylic acid (TCA) cycle intermediate, α-ketoglutarate (α-KG) [29]. In turn, Glu released from neurons can be transported to the astrocyte *via* glutamate transporters [see below], where it is amidated to Gln, completing the metabolite shuttling between astrocyte and neurons, referred to as the glutamine/glutamate-GABA cycle (GGC). Several enzymes which are necessary to ensure CNS glutamate and GABA homeostasis are heterogeneously distributed among neurons and astrocytes [37]. The glutamine synthesizing enzyme, GS, is exclusively localized in astrocytes [38], although few studies have reported its expression in both oligodendroglia and microglia under pathological conditions [39,40]. Studies in neuron-astrocyte co-cultures demonstrated that GS expression in the latter is positively regulated by neurons, and this effect appears to be mediated by neuron-derived Glu or trophic factors, requiring direct contact between astrocytes and nerve cell matrix [41,42]. An *in situ* study suggested that in the cerebellum phosphate-activated glutaminase (PAG) is primarily located in neurons rather than astrocytes [43], although a low activity of this enzyme has been observed in cultured astrocytes [43,44].

The metabolic neuronal-astrocytic interaction is well demonstrated by the dependence of neurons on astrocyte-derived thiols for optimal maintenance of stable concentrations of GSH [45]. GSH, the main antioxidant constitutes ~90% of the intracellular non-protein thiols and plays a prominent role in the detoxification of ROS and neutralization of organic hydroperoxides. It is noteworthy that GSH levels are lower in neurons than in astrocytes [46] and that cysteine derived from astrocytes is essential for the maintenance of stable GSH levels in neurons. In general, neuronal stores of GSH are largely dependent upon astrocytic stores, and neurons are more vulnerable than astrocytes to oxidative stress. Several co-culture studies have elegantly demonstrated that astrocytes protected neurons from toxicity by a GSH-dependent mechanism [47,48].

Astrocytes also play a pivotal role in neurometabolic coupling, referred to as the astrocyte neuronal lactate shuttle hypothesis (ANLSH). ANLSH invokes that glucose enters the CNS *via* astrocytic processes [49]. ANLSH involves glutamate-stimulated aerobic glycolysis and uptake of Glu by astrocytes. The ensuing activation of the Na^+^-K^+^-ATPase triggers glucose uptake and processing *via* glycolysis, thus mediating release of lactate from astrocytes [50]. Lactate can be shuttled into neurons where it is used to meet their energy demand and works as a potent neuroprotective agent [51]. More recently, the alternative neuron–astrocyte lactate shuttle (NALS) hypothesis was developed, suggesting that depending on the thermodynamic and kinetic status of the cytosolic and mitochondrial redox states, lactate transfers from neurons to astrocytes [52]. In general, studies support the notion that neurons have the highest energy demand of all neural cells and are most vulnerable to energy failure [53]. It is important to note that recent modeling studies of astrocyte-neuron metabolic interactions have minimized the role of lactate as a major energy agent, suggesting that neurons are also capable of utilizing different fuel sources for incorporation into metabolic pathways, such as glucose, ketone bodies and fatty acids [54].

Intermediate filament proteins expressed in astrocytes, such as glial fibrillary acidic protein (GFAP) and vimentin, are involved in processes by which astrocytes control neurogenesis as well as other aspects of neural plasticity and regeneration. The ablation of GFAP and vimentin increases axonal and synaptic regeneration in mice [55-58]. Furthermore, thrombospondins extracellular matrix proteins secreted by astrocytes have been shown to promote synaptogenesis during development as well as in experimental mouse models of stroke [59].

It is noteworthy that astrocytes also play a dynamic role in CNS function by maintaining the restrictive properties of the blood–brain barrier (BBB). As an essential neurovascular component, astrocytes directly regulate the properties of this barrier. Furthermore, astrocytes can regulate the neurogenic niche indirectly by regulating the accessibility of blood-borne factors/molecules which are involved in neurogenesis [60].

Finally, astrocytes release factors that sustain neuronal function and viability. Astrocytes synthesize and secrete a wide range of neurotrophic and growth factors, cytokines, extracellular matrix proteins, proteoglycans and cholesterol which are involved in neuronal survival, proliferation, differentiation and synaptogenesis [61,62].

#### Astrocytes and manganese in some neuropathological conditions: focus on Alzheimer’s disease (AD) and chronic hepatic encephalopathy (HE)

Astrocytes play a critical role in the progression and outcome of neuropathological processes by reducing neural damage and promoting the revascularization of the surrounding tissue through reactive astrogliosis and neuroinflammation, the latter characterized by secretion of pro-inflammatory factors, such as interleukins [62-66]. On the other hand, reactive astrogliosis has been shown to be associated with functional impairment of astrocytes, and diminished neuronal support by astrocytes has been invoked in multiple neuropathological conditions. For example, in mice expressing human mutant Tau, a model of neurological disorders, an increase of GFAP-immunoreactive astrocytes and neuronal loss were shown in the spinal cord [67]. Other studies have implicated astrocyte-mediated Glu recycling in the pathogenesis of Alzheimer’s disease (AD) [68]. Impairment in astrocytic Glu transport and reduction in the expression of the Glu transporters, L-glutamate-L-aspartate transporter (GLAST) and glutamate transporter 1 (GLT-1) has been invoked in a mouse model of Tau pathology. In homogenates of AD cortex, GFAP and GLAST expression were shown to inversely correlate with increased expression of GFAP and down regulation of GLAST as well as increased Braak stage [69]. Additionally, major disruption in Gln metabolism has been shown in AD. For example, GS activity in cortical homogenates from AD brain was found to be lower in comparison to homogenates derived from non-demented brains [70]. Moreover, the concentration of Glu was shown to be elevated, while the concentration of Gln was lower in cerebrospinal fluid of AD patients [71].

It is important to note that Mn was shown to induce cell swelling in cultured astrocytes and that astrocytic pathology, such as gliosis and Alzheimer type II astrocytes were observed in both animal and cell culture models in response to manganese exposures [72,73]. Morphologic changes of astrocytes are also a major feature of hepatocerebral disorders, including chronic hepatic encephalopathy (HE) [74]. Hepatic encephalopathy is a clinical complication of liver failure with a wide spectrum of neuropsychiatric complication. Glial cells were described as a primarily target in HE and as a cell responsible for the neuronal pathology [75]. The most prominent histopathological changes found in HE associated with chronic liver failure include Alzheimer’s type II astrocytosis and astrocytic swelling, leading to brain edema [75-77]. Notably, Mn was found to be a significant etiologic factor in low–grade brain edema observed in HE, affecting the dopaminergic neuronal system and dopaminergic receptor activity [73,78]. Interestingly, Mn and another important etiologic factor in HE, namely ammonia, cause similar morphologic and functional changes in astrocytes. Both are potent glial toxins and have the capacity to act synergistically to activate the mitochondrial benzodiazepine receptor leading to increased synthesis of neurosteroids and GABAergic signaling [79]. Moreover Mn and ammonia downregulate the astrocytic Glu transporters, leading to impairment in neurotransmission [80].

### Manganese involvement in astrocyte function

Brain Mn is preferentially deposited in astrocytes given the presence of high-capacity transporters within these cells. The concentration of Mn in astrocytes is 50–60 higher than in neurons [81]. At the subcellular level, the highest Mn concentration in astrocytes is noted within mitochondria [82]. Mn causes oxidative stress in primary cultures of astrocytes, leading to the mitochondrial dysfunction and energy insufficiency [22,83]. One of the possible mechanism of Mn-dependent failure of astrocytes to maintain antioxidant defence mechanisms is disruption of glutathione (GSH) synthesis [72,84,85]. In addition, Mn has a similar effect on taurine in both neurons and co-cultures; the latter also serves as a free radical scavenger and important neuroprotective amino acid [86].

Brain energy metabolism depends almost exclusively on the oxidation of glucose [87]. Glucose metabolism leads to the biosynthesis of neurotransmitters such as Glu, aspartate, and GABA [38]. Mn causes metabolic changes in astrocytic glucose metabolism by inhibition of the astrocyte-specific enzyme, GS [88,89]. Mn also plays an important role in brain energy metabolism by affecting the key anaplerotic, glial-specific enzyme, PC [90,91]. These Mn-induced effects on astrocytic pathology may cause a dyshomeostasis between neurons and astrocytes, leading to further neuronal synaptic dysfunction and activation of an excitotoxic state.

Mn was also shown to induce the expression of astrocytic inflammatory products as well as signal transduction mediators [72,92]. A recent study has shown increased transport of the nitric oxide (NO) substrate, L-arginine in Mn exposed astrocytes [93]. Other reports confirmed that Mn affects astrocytic inducible nitric oxide synthase (iNOS) expression and NO production upon activation of C6 glioma cells with LPS/ TNFγ or primary astrocyte cultures with TNFα/TNFγ [94,95]. Astrocytic exposure to Mn is also associated with cell swelling secondary to NOS activation. Moreover, increased expression of the water channel protein aquaporin-4 (AQP4), a predominant astrocytic isoform, has been implicated in Mn-mediated cell swelling [96].

As mentioned above, astrocytes support multiple neuronal functions through multiple processes; thus Mn-mediated disturbances in astrocytes function would be expected to cause neuronal demise. Indeed, an *in vivo* study revealed that Mn-mediated neuronal injury in the striatum and the globus pallidus is associated with primary dysfunction of astrocytes via mechanisms involving NO [94]. An I*n vitro* study demonstrated that Mn inhibits the ability of astrocytes to promote neuronal differentiation by a mechanism that involves oxidative stress and a reduction in levels of the extracellular matrix protein, fibronectin [97].

### Glutamine in the central nervous system

Gln abounds in the CNS, and its concentrations are at least one order of magnitude higher than any of the other amino acid in the interstitial and cerebrospinal fluid (CSF) [98]. Gln plays a prominent role in general CNS metabolism by supporting tissue homeostasis. As discussed above, Gln is the amino acid that directly couples ammonia metabolism to the synthesis of the amino acid neurotransmitter Glu, and indirectly to GABA. This reaction requires active communication between neurons and astrocytes, and is accomplished by the GGC cycle. The major role of the GGC is to thwart the extracellular Glu levels, thus preventing excitotoxicity. Moreover, Gln cycling between neurons and astrocytes produces compounds that are direct precursors of the tricarboxylic acid (TCA) cycle intermediate α-ketoglutarate, thus maintaining the high demand for energy within the brain [29].

Transport of Gln across the membranes of CNS cells is mediated by multiple transport systems, characterized by their overlapping substrate specificities, substrate affinities and cellular distribution [36]. Among these systems, the sodium-dependent systems ASC, A and N play dominating roles in Gln turnover. Immunocytochemical analysis and Gln transport measurements both *in vivo* and *in vitro* revealed the compartmentation of Gln-transporting proteins between astrocytes and neurons. The bi-directional system N transporters SNAT3 or SNAT5 which catalyze Gln transport, are exclusively located within astrocytes. SNAT3 is believed to specifically mediate Gln efflux from astrocytes [99,100]. In addition to System N, release of Gln from astrocytes is mediated by other transport systems, such as the sodium-independent System L (LAT2) and the sodium-dependent System ASC (ASCT2) [98,101,102]. The unidirectional system A transporter SNAT1, which catalyzes Gln uptake, is predominately expressed in neurons [98]. A portion of astroglial-derived Gln across the BBB to the periphery is mediated by system N transporters along with some additional contribution from the System L transporters [103].

### Manganese and glutamine turnover

The Gln/Glu-GABA cycle represents a complex process, since Gln efflux from astrocytes must be met by its influx in neurons. Mn toxicity is associated with the disruption of both of these critical points in the GGC. In cultured astrocytes, pre-treatment with Mn inhibits the initial net uptake of Gln in a concentration-dependent manner [83]. Mn added directly to astrocytes induces deregulation in the expression of SNAT3, SNAT2, ASCT2 and LAT2 transporters [83]. Corroborating the changes in transporter protein expression levels, astrocytes treated with Mn displayed a significant decrease in Gln uptake mediated by the transporting Systems N and ASC, and a decrease in Gln efflux mediated by Systems N, ASC and L (Table 1) [104].

**Table 1:** Manganese involvement in glutamine/glutamate-GABA cycle: disruption of glutamine and glutamate transporters

**Name of transporter**		**Changes down (down-regulation) mediated by manganese**	**Cellular localization**	**References**
Glutamine transporter	System			
SNAT3	System N	mRNA and protein expression; function(uptake and efflux)	astrocytes	[67], [81]
SNAT2	System A	mRNA and protein expression	astrocytes	[81]
ASCT2	System ASC	protein expression; function (uptake and efflux)	astrocytes	[81]
LAT2	System L	mRNA and protein expression; function (uptake)	astrocytes endothelial cells	[81]
Glutamine transporter				
GLAST		protein expression; function (uptake)	astrocytes	[98], [103]
GLT-1		mRNA and protein expression; function (uptake)	astrocytes	[98], [103]

**Figure 1:**
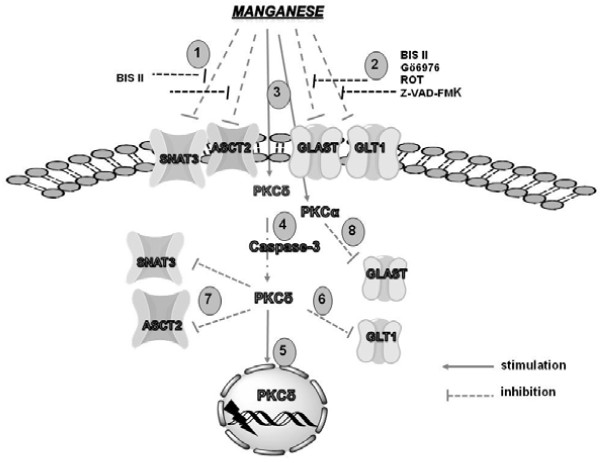
**A proposed model describing the mechanisms of Mn-mediated disruption of glutamine/glutamate-GABA cycle.** Mn exposure leads to the deregulation of Gln uptake by dysfunction of SNAT3 and ASCT2 activity, while incubation with BIS II (bisindolylmaleimide, general PKC inhibitor) reverses this effect (1); Mn exposure leads to the deregulation of Glu uptake by dysfunction of GLT1 and GLAST activity, while incubation with BIS II, Gö6976 (PKCα inhibitor) , ROT (rottlerin, PKCδ inhibitor) and Z-VAD-FMK (Z-Ala-Glu(OMe)-Val-Asp(OMe)-fluoromethyl ketone caspase inhibitor) reverses this effect (2); Mn exposure leads to the activation of PKCδ and PKCα (3); additionally, Mn mediates activation of PKCδ by caspase-3-dependent proteolytic cleavage (4); proteolytically activated PKCδ translocates to the nucleus and mediates DNA fragmentation (5); Mn-mediated disruption of SNAT3, ASCT2 and GLT1 expression involves PKCδ signalling (6,7); GLAST expression is down regulated by Mn *via* PKCα signalling (8).

The contribution of PKC signalling to Mn-induced dyshomeostasis in Gln transport has been investigated in cultured astrocytes. A recent study revealed that PKC inhibition by its general inhibitor bisindolylmaleimide II (BIS II) effectively reversed the Mn-induced down-regulation in Glu uptake. Treatment of primary astrocyte cultures with a PKC stimulator caused decreased Gln uptake mediated by Systems ASC and N, and decreased expression of ASCT2 and SNAT3 protein levels in cell lysates and in plasma membranes [105]. It is noteworthy that both transporters contain putative PKC phosphorylation sites, conserved in the human, rat and mouse [106,107]. In addition, a recent *in situ* study showed that PKC activation induced phosphorylation and internalization of SNAT3 [108]. Furthermore, increased binding of PKCδ to ASCT2 and SNAT3 upon exposure to Mn has been identified by co-immunoprecipitation. In contrast, Mn exposure causes increased phosphorylation of PKCδ in cultured astrocytes. Taken together, these findings suggest a prominent role of PKCδ in Mn–mediated disruption of Gln turnover (Figure 1).

### Ubiquitin-mediated proteolytic system in Mn-mediated disruption of SNAT3

A recent study revealed that the astrocytic transporter SNAT3 possesses the highest affinity/specificity to Gln among of all the investigated carriers, and that it is the most sensitive to Mn exposure. This transporter is readily degraded subsequent to a relatively short exposure to Mn [104]. In addition, it was suggested that the ubiquitin-mediated proteolytic system might be involved in the Mn-mediated down-regulation of SNAT3 [105]. Mn exposure has been noted to increase both free ubiquitin levels and ubiquitinated proteins in primary cultures of astrocytes. Furthermore, we recently showed a selective interaction of SNAT3 with the ubiquitin ligase, Nedd4-2 (neuronal precursor cell expressed, developmentally down-regulated 4–2) upon Mn exposure in astrocytes [105]. It is noteworthy that Nedd4-2 mediates the down-regulation of numerous membrane channels and transporters *via* ubiquitination and subsequent degradation by the proteasome [109]. Polyubiquitination plays a key role in transporter sorting and targeting for lysosomal degradation, which also involves the proteasome [110]. A recent study has confirmed that serum- and glucocorticoid-induced kinase 1 (SGK1) phosphorylates the ubiquitin ligase Nedd4-2, decreasing the interaction between Nedd4-2 and target proteins [111]. Furthermore, in a *Xenopus laevis* oocyte expression system it has been suggested that SNAT3 is down regulated by Nedd4-2, and that this effect is restored by SGK1 [112]. Notably, Mn exposure leads to decreased SGK1 expression and phosphorylation, suggesting Nedd4-2/SGK1 involvement in Mn-mediated degradation of SNAT3. These findings, concomitant with evidence that chronic Mn exposure alters the expression of genes associated with the ubiquitin/proteasome system [113], suggest that Mn disrupts SNAT3 expression or function by promoting its ubiquitination [105].

It has also been shown that PKC activation results in the ubiquitination of the dopamine transporter (DAT), leading to its internalization and degradation [114,115]. Furthermore, the knockdown of Nedd4-2 resulted in a dramatic reduction in the PKC-dependent ubiquitination of DAT [115]. PKC activation also led to hyper-ubiquitination, and increased the interaction between SNAT3 and Nedd4-2. PKC stimulation caused a decrease in astrocytic Gln uptake mediated by System N and significantly decreased SNAT3 protein levels, while Mn exposure activated PKCs [82]. Taken together, these findings demonstrate that Mn-induced deregulation of SNAT3 function is likely mediated *via* PKC signalling and accompanied by an increase in ubiquitin-mediated proteolysis.

### Manganese and glutamate transport

Glu is the prominent excitatory neurotransmitter in the mammalian CNS [116]. Regulation of synaptic Glu concentrations is critical to normal CNS function. Glu released into the synaptic cleft in taken up by Glu transporters mainly present perisynaptically on astrocytes. Only a small amount is taken up by presynaptic neurons. Astrocytes take up glutamate *via* the sodium-dependent Glu transporters, GLAST, GLT-1 and the sodium-independent chloride-dependent Glu-cystine antiporter [29]. Accordingly, astrocytes regulate the levels of extracellular Glu and influence synaptic activity. Using antisense knockdown or pharmacological inhibition of Glu transporters, it has been demonstrated that disruption of transporter function increases the vulnerability of neurons to excitotoxic insults [31]. The functional relevance of the Glu transporters was demonstrated in animal studies, where knockout of GLAST resulted in impaired performance on an accelerating rotarod, and partial loss of GLT-1 led to hind limb paralysis [117,118]. Impairment in astrocyte-mediated recycling of Glu represents the major contributing factor to neuropathology, and it has been invoked in the etiology of several neurodegenerative diseases, including AD, Parkinson disease (PD) and amyotrophic lateral sclerosis (ALS) [119].

Several studies established that Mn disrupts Glu transporting systems leading to both a reduction in Glu uptake and elevation in extracellular Glu levels [120] (Table 1). For instance, Chinese hamster ovary (CHO) cells transfected with GLAST or GLT-1 show impairment in Glu transport in response to Mn exposure [121]. Long-term airborne Mn exposure leads to the down regulation of GLAST and GLT-1 In non-human primate brain, both at the mRNA and protein levels [84]. Although the mechanisms of Mn–mediated disruption in transporter expression have yet to be completely understood, lysosomal proteolysis has been implicated in GLT-1 degradation upon exposure to this metal [122,123].

PKC signalling has been implicated in Mn-induced down-regulation of Glu turnover in primary astrocyte cultures [124]. A recent study revealed that PKC stimulation by α-phorbol 12-myristate (PMA) significantly decreased astrocytic Glu uptake, while treatment with the general PKC inhibitor BIS II, reversed the Mn-induced down regulation of Glu transport. Moreover, Mn-dependent down-regulation in astrocytic Glu uptake was reversed by specific inhibitors of PKCδ - rottlerin (ROT), and PKCα- Gö6976 or caspase 3 inhibitor-Z-Ala-Glu(OMe)-Val-Asp(OMe)-fluoromethyl ketone (Z-VAD-FMK) [122]. Similarly, Mn-induced down-regulation of GLT1 protein level was reversed by Bis II, ROT, Gö6976 and Z-VAD-FMK inhibitors. Mn-dependent down-regulation of astrocytic GLAST expression was also attenuated in the presence of PKCα and casapase-3 inhibitors. Furthermore, direct association between GLT-1, but no GLAST, and the PKCδ or PKCα isoforms, and Mn-induced increases in PKCδ-GLT-1 interaction have been noted in a co-immunoprecipitation study [122].

The role of the PKCδ isoform in Mn-induced deregulation of Glu turnover was estimated by a knockdown study. Astrocytes transfected with shRNA against PKCδ, (but not with PKCα) showed lessened sensitivity to Mn compared to those transfected with control shRNA, suggesting a predominant role for the PKCδ isoform in Mn-dependent down-regulation of Glu turnover [124]. Parallel to these observations, recent evidence shows that PKC signalling is critical in Glu transporter regulation [104]. PKC-mediated decrease in Glu transporter function may involve changes in transporter activity or number/expression by increasing the rates of endocytosis or decreasing the redistribution of the transporters from a subcellular compartment to the plasma membrane. Another possible mechanism of PKC-induced negative regulation of Glu transporters invokes down-regulation of transporter activity at the cell membrane. For example, in C6 glioma cells transfected with GLT-1 and in primary cultures endogenously expressing GLT-1, activation of PKC rapidly down regulates GLT-1 cell surface expression [124]. As far as GLAST transporter function, several groups found contradictory results, showing decreases as well as increases in GLAST function in response PKC activation [125]. Several studies using cell cultures as well as animal models indicate that proteolytic activation of PKCδ by caspase-3 plays a major role in the regulation and execution of apoptosis [126]. In addition, previous studies showed that caspase-3-dependent PKCδ activation not only contributes to neuronal apoptosis, but also has a regulatory role in amplification of the apoptotic cascade during neurotoxic stress inherent to Mn treatment *via* positive feedback loop between PKCδ and caspase-3 activation [127]. Proteolytic activation of PKCδ promotes its nuclear translocation and PKCδ-dependent mediation of DNA fragmentation. Notably, Mn toxicity is associated with caspase-3 activation in several *in vitro* and *in vivo* models [127-130] as well as increased PKCδ’s nuclear translocation [124]. Co- treatment with the PKCδ inhibitor ROT or the caspase-3 inhibitor Z-DEVD-FMK blocked Mn-induced DNA fragmentation in mesencephalic dopaminergic neuronal [N27] cells. Additionally, N27 cells expressing a catalytically inactive PKCδ protein or a caspase-3 cleavage resistant PKCδ protein were found to be resistant to Mn-mediated apoptosis [127].

A mechanism implicating caspase-3 and PKCδ inhibition has been invoked for the Mn-mediated disruption of Glu and Gln transport in astrocytes [124]. Collectively, activation of PKC signalling seems to be a ubiquitous mechanism for the regulation of GGC by Mn (Figure 1).

## Review and Conclusion

Astrocytes are involved at multiple levels in brain (patho)physiology *via* their interaction with neurons. The role of astrocytes in neuronal activity and survival is well illustrated in pathological conditions mediated by disruption of the Gln/Glu-GABA cycle. The deregulation of Gln homeostasis may consequently diminish the availability of this amino acid to neurons. These effects are posited to lead to impairment of Glu-ergic neurotransmission and are very well recognized in Mn neurotoxicity. The findings reported in this review suggest that a better understanding of Gln cycling between neurons and astrocytes may provide knowledge about normal brain function and highlight potential molecular tools for therapeutic interventions in pathology caused by Mn.

## Competing interests

The authors declare that they have no competing interests.

## Authors’ contributions

MSW carried out several of the studies that are reviewed herein and was the lead author, synthesized the literature and was involved in drafting the manuscript. MA provide conceptual input and participated in the coordination. All authors read and approved the final manuscript.

## Pre-publication history

The pre-publication history for this paper can be accessed here:

http://www.biomedcentral.com/2050-6511/14/23/prepub
